# Stabilized nanosystem of nanocarriers with an immobilized biological factor for anti-tumor therapy

**DOI:** 10.1371/journal.pone.0170925

**Published:** 2017-02-06

**Authors:** Angelika Kwiatkowska, Ludomira H. Granicka, Anna Grzeczkowicz, Radosław Stachowiak, Michał Kamiński, Zuzanna Grubek, Jacek Bielecki, Marcin Strawski, Marek Szklarczyk

**Affiliations:** 1 Nałęcz Institute of Biocybernetics and Biomedical Engineering Polish Academy of Sciences, Warsaw, Poland; 2 Department of Applied Microbiology, Faculty of Biology, University of Warsaw, Warsaw, Poland; 3 Laboratory of Electrochemistry, Faculty of Chemistry, University of Warsaw, Warsaw, Poland; Brandeis University, UNITED STATES

## Abstract

**Objective:**

The inadequate efficiency of existing therapeutic anti-cancer regiments and the increase in the multidrug resistance of cancer cells underscore the need to investigate novel anticancer strategies. The induction of apoptosis in tumors by cytotoxic agents produced by pathogenic microorganisms is an example of such an approach. Nevertheless, even the most effective drug should be delivered directly to targeted sites to reduce any negative impact on other cells. Accordingly, the stabilized nanosystem (SNS) for active agent delivery to cancer cells was designed for further application in local anti-tumor therapy. A product of genetically modified *Escherichia coli*, listeriolysin O (LLO), was immobilized within the polyelectrolyte membrane (poly(ethylenimine)|hyaluronic acid) shells of ‘LLO nanocarriers’ coupled with the stabilizing element of natural origin.

**Methods and results:**

The impact of LLO was evaluated in human leukemia cell lines *in vitro*. Correspondingly, the influence of the SNS and its elements was assessed *in vitro*. The viability of targeted cells was evaluated by flow cytometry. Visualization of the system structure was performed using confocal microscopy. The membrane shell applied to the nanocarriers was analyzed using atomic force microscopy and Fourier transform infrared spectroscopy techniques. Furthermore, the presence of a polyelectrolyte layer on the nanocarrier surface and/or in the cell was confirmed by flow cytometry. Finally, the structural integrity of the SNS and the corresponding release of the fluorescent solute listeriolysin were investigated.

**Conclusion:**

The construction of a stabilized system offers LLO release with a lethal impact on model eukaryotic cells. The applied platform design may be recommended for local anti-tumor treatment purposes.

## Introduction

Modern medicine undoubtedly can boast great successes in the control of tumor development and progression. Nevertheless, an increase in cancer-related mortality is still observed in developed countries [1–4]. This increase is caused by both side effects associated with the conventional treatment (e.g., surgery, radiotherapy and chemotherapy) and low efficiency of used drugs, which are increasingly proven to be inadequate [5]. Furthermore, only a small amount of an active factor is delivered directly to the targeted cells due to significant losses during transport. Accordingly, it is often necessary to use an excess of applied medicines. Unfortunately, the vast majority of cytostatic agents are dissipated through the body, causing significant emaciation of patient organisms. As a consequence, the benefits obtained from the application of antitumor factors are overshadowed by numerous excessive side effects. The most frequently occurring afflictions recorded during clinical use of typical chemotherapeutic agents include bone marrow suppression, heart failure (as a consequence of cardiomyopathy) typhlitis and dyspigmentation [6–9]. In recent decades, considerable efforts have been made to change this state of affairs and reduce the undesirable effects that are connected with classic anti-tumor therapies.

Recently, novel anti-tumor agents with highly specific mechanism of actions have been discovered [10–11] and existing strategies targeting the delivery of cytostatic factors to cancer cells have been developed to reduce the unwanted side effects of the treatment and improve its safety. For these reasons, various platforms for drug transport have been intensively examined. In particular, systems with a core coated with polymeric membrane shells encompassing an active agent have been previously widely described. Scientists have applied different materials for the production of cores of this type, including gold or gold nanocomposite nanoparticles [12], magnetic nanoparticles [13–16], silica particles [17] and polystyrene cores [18–19]. Particles like poly(lactic acid) [20] or poly(L-lactide)–poly(ethylene glycol) particles [21], carbonate cores [22–23], starch-based nanoparticles [24], nanocomposite nanoparticles [13, 25–26] and even cells as sacrificial cores [27–28] were also applied.

It should be noted that kernels of natural-origin attract special attention in the field of active agent delivery because of their certain properties, such as a cell-like size and high ligand binding ability. Furthermore, in combination with polyelectrolyte membranes, natural cores can build systems with high biocompatibility.

Despite the numerous examined medicines and drug delivery systems, an optimal system that exhibits efficiency as an anti-tumor therapy with reduced side effects has not been yet obtained. An application of factors inducing tumor necrosis might introduce a major change in this area. However, in the majority of the proposed systems, drug molecules are released into the medium surrounding the tumor tissues and subsequently are adsorbed by healthy cells. A different approach to combat this issue involves the process of adsorption of the whole cytostatic carriers. The success of such a strategy is determined by the size of the carriers, which can be internalized via phagocytosis, and the efficiency of the anti-tumor factor immobilized within the carrier.

We have described the stabilized nanosystems (SNS) based on elements of natural origin as the platform for antitumor factor delivery to the targeted cells. The main parts of the designed platform are 200-nm diameter preserved bacterial carriers that bear a cytostatic agent—listeriolysin O (LLO) [29–32]. The size of the applied carriers ensures their easy absorption by tumor cells via phagocytosis. Moreover, to reduce active agent destruction during the experiment, it has been immobilized within the nanothin, biocompatible polyelectrolyte (PE) membrane-shell covered carriers. To stabilize the system and increase its anti-tumor efficiency, the constructed LLO nanocarriers were coupled with the stabilizing element, namely the cell core, via a biotin-avidin-biotin bridge. Additionally, the surface of the stabilizing core was modified by transferrin complex to provide system selectivity. We evaluated the impact of the designed system *in vitro* in human peripheral blood mononuclear cells or Jurkat, WEHI-164 and IC-21 cell lines. The obtained results prove that our system allowed the cytostatic release in eukaryotic cells by exerting a lethal impact.

Herein, we report the first platform for local LLO delivery in which bacterial nanocarriers are coupled with naturally derived stabilizing elements. The application of mostly natural elements is the unique feature of our platform. The membrane construction applied in the present system ensures the increase of the system avidity towards tumor cells. Thus, the SNS provides specific delivery of the cytostatic factor to the targeted cells and simultaneously reduces the number of potential side effects caused by the anti-tumor therapy.

## Materials and methods

### Physicochemical characterization of polyelectrolyte shells

#### Spectroscopic evaluation of polyelectrolyte shells

The polyelectrolyte (PE) membrane on a substrate was analyzed by Fourier transform infrared spectroscopy (FTIR) (4000–666 cm^−1^) at the beginning of the experiment. The examination was performed using FTS 3000MX spectrometer (Bio-Rad Excalibur, Cambridge, MA, USA). Liquid samples were collected in a KBr pellet. Typically, thirty scans were performed at a resolution of 4 cm^−1^ and selectivity of 2 cm^−1^. Presented FTIR curves were analyzed using Essential FTIR software (FTIR Varian Resolution Pro 4.1.0.101, Randolph, MA, USA).

#### Atomic force microscopy evaluation of polyelectrolyte shells

The surface morphology of the samples was imaged using Nanoscope 8 AFM microscope with a J scanner (Bruker, USA). PeakForce Tapping^®^ mode was applied during examination. Scratching procedure for film thickness determination was described previously [33]. Then, polyelectrolyte layers were visualized in the 2D or 3D form using Nanoscope software. All of the images were obtained at room temperature.

For surface forces acquisition, the silicon cantilever with a borosilicate glass colloidal particle of a 10 μm diameter were used (SQube, Germany). Spring constant value of a used cantilever was determined before experiment with ThermalTune method. The force-distant data were acquired in Nanoscope 8.15 software and analyzed in Origin 8.50 (OriginLab).

#### Evaluation of the wettability angle of polyelectrolyte shells

The surface wettability angle of the applied polyelectrolyte membrane was analyzed using a surface energy analyzer (HAAS, UE) with dedicated software.

### Design of the systems for active agent delivery

#### Construction and synthesis of GPF-LLO

To obtain the GFP-LLO fusion and control proteins, the *hly* gene sequence from *Listeria monocytogenes* 10403S chromosome and the *gfpmut3b* sequence were PCR amplified and fused to OE-PCR using specific oligonucleotides. The resulting *hly*, *hly-gfp* and *gfp-hly* genes were cloned into the pPSG-IBA series plasmids (which allows attachment of the 6xHistidine-tag to the fusion protein and expression from the bacteriophage T7 promoter) using the StarGate Cloning System (IBA BioTagnology, Goettingen, Germany). Then, the recombinated pPSG-IBA plasmid was transformed into the *E*. *coli* BL21(DE3) production strain. The LLO, LLO-GFP and GFP-LLO proteins were purified from the bacterial cell lysates using Ni-NTA resin columns via affinity chromatography and concentrated with a centrifugal concentrator. Construction and purity was confirmed by SDS PAGE and western blot, and activity was assessed using the hemolytic test [34]. The final concentration 0.6 μg/ml was estimated by NanoDrop spectrophotometer.

#### Immobilization of GFP-LLO within the polyelectrolyte

GFP-LLO prepared according to the procedure described above was dissolved in 0.1 M NaCl at pH 7.2 in 1:2 (v/v) ratio (GFP-LLO:NaCl). Then, hyaluronic acid (HA) (Sigma, EU) was dissolved in 0.1 M NaCl to obtain a final concentration of 1 mg/ml at pH 7.2, whereas biotinylated hyaluronic acid solution (HA_biot_) was prepared according to the previously described procedure [35]. Finally, both HA or HA_biot_ solutions were mixed with GFP-LLO in 1:1 (v/v) ratio to obtain HA+GFP-LLO or HA_biot_+GFP-LLO, respectively.

#### Coating of the bacterial core with polyelectrolytes to obtain LLO nanocarriers

Poly(ethylenimine) (PEI) (MW 60 kD, Aldrich, USA) was dissolved in 0.1 M NaCl to obtain a concentration of 1 mg/ml at pH 7.2. The suspension of preserved bacterial cells at concentration 1×10^8^ cells/ml was incubated with PEI solution for 4 minutes. Then, bacteria were washed twice in RPMI-1640 (Biomed, UE) at 1000 rpm for 3 minutes to remove unabsorbed polyelectrolyte. The same procedure was repeated with the HA+GFP-LLO solution described above. Finally, ‘LLO nanocarriers’ (bacteria coated with PEI and HA+GFP-LLO—bacteria|PEI|HA+GFP-LLO) were obtained. Moreover, an additional platform was prepared in which HA_biot_ was applied instead of the HA layer (bacteria|PEI|HA_biot_+GFP-LLO). Simultaneously, the adequate systems (negative controls) without LLO were prepared, including bacteria|PEI|HA and bacteria|PEI|HA_biot_.

#### Modification of ‘nanocarriers’ with ligands

‘LLO nanocarriers’ were incubated for 15 minutes with a biotin solution (Sigma, USA) at a concentration of 0.2 mg/ml in 0.1 M NaCl at pH 7.2 followed by washing. The biotinylated ‘LLO nanocarriers’ were stirred with 0.2% avidin solution (Sigma, USA) in 0.1 M NaCl at pH 7.2 to obtain the biotinylated nanocarriers complexed with avidin. After washing, the modified carriers were incubated in a solution of biotinylated human transferrin (Sigma, USA) at concentration 1 mg/ml in 0.1 M NaCl at pH 7.2 followed by washing in phosphate-buffered saline (PBS) (Biomed, UE). Finally, ‘ligand modified LLO nanocarriers’ (bacteria|PEI|HA+GFP-LLO+TR) were obtained. Simultaneously, the adequate system (negative control) without LLO was prepared, namely bacteria|PEI|HA+TR.

#### Preparation of the stabilizing element of the system (‘cell core’)

WEHI-164 cells at concentration 0.5×10^6^ were preserved in ethanol, washed twice with PBS and incubated for 15 minutes with 0.2 mg/ml (w/v) biotin solution in 0.1 M NaCl at pH 7.2 followed by washing. Then, the biotinylated cells were stirred with 0.2% avidin solution in 0.1 M NaCl at pH 7.2, resulting in biotin-avidin complex formation on cells. After washing, the cells were mixed with 1 mg/ml biotinylated transferrin solution in 0.1 M NaCl at pH 7.2 and rinsed in PBS after incubation. Thus, the stabilizing element (‘cell core’) of the designed system was obtained.

#### Design of the stabilized nanosystem (SNS)

‘Cell cores’ mentioned above were incubated with previously prepared biotinylated ‘LLO nanocarriers’ (bacteria|PEI|HA_biot_+GFP-LLO) for 15 minutes followed by washing. Accordingly, the stabilized nanosystem (SNS) was obtained. The system was built from the ‘cell core’ linked with biotinylated ‘LLO nanocarriers’ through avidin-binding sites (not joined with biotinylated transferrin).

### Evaluation of the trace of designed platform interaction with targeted cells

#### Coating of model particles (of 200 nm) with polyelectrolytes

To facilitate system visualization, non-fluorescent bacterial nanocarriers in the SNS structure were replaced by fluorescent microbeads (FITC labeled beads 200 nm in diameter (Microprobes, USA)).

The suspension of microbeads was incubated with PEI solution (prepared accordingly to the procedure described above) in a 1:100 (v/v) ratio (microbeads:PEI) for 4 minutes. Afterward, microbeads were washed twice in RPMI-1640 at 1000 rpm for 3 minutes to remove unabsorbed polyelectrolyte. The same procedure was repeated with a solution of HA. Microbeads coated with a PEI|HA bilayer were obtained (μB).

#### Evaluation of the trace of 200-nm carrier interaction with targeted cells

The μB were added to WEHI-164 cells culture at a concentration of 0.5×10^6^ cells/ml in 1:50 (v/v) ratio (μB:WEHI-164). Targeted cells were maintained (37°C, 5% CO_2_) in culture medium RPMI-1640|10%NCS (Biochorom, EU) for 24 hours. The cells without the addition of the microbeads were cultured (37°C, 5% CO_2_, in culture medium RPMI-1640/10% NCS) as a negative control. The percentage of cells exhibiting fluorescence was evaluated after 2- or 24-hour culture using flow cytometry.

#### Visualization of the structure of the SNS

Confocal microscopy was applied for the visualization of the designed structure of the SNS. FITC-labeled microbeads 200 nm in diameter were coated with the PEI|HA bilayer (μB) that was prepared according to the procedure described above and applied as a bacterial core model in these studies. The ‘cell core’ was dyed with Hoechst 33342 (Invitrogen, USA).

Imaging was performed on an FV1000 system with spectral detectors (Olympus) using a 60x/1.20 water immersion objective lens. An argon-ion laser was applied. Images were processed using the FluoView and Fiji software.

### Evaluation of the designed systems impact on cells

#### Cell line culture

Jurkat human leukemia T-lymphocyte cells (ATCC, Rockville, MD, USA), WEHI-164 cells (ATCC, Rockville, MD, USA) and IC-21 mouse macrophage cells were cultured in RPMI-1640 media supplemented with 10% newborn calf serum (NCS) (Biochorom, EU) and 1% penicillin and streptomycin as selective antibiotics. Non-adherent cells (Jurkat) were passaged every third day by diluting to a final concentration of approximately 0.5×10^6^. The cells grew at 37°C in an atmosphere of 5% CO_2_.

Adherent cells WEHI-164 or IC-21 were cultured to greater than 90% confluence and then washed with Dulbecco’s Phosphate Buffered Saline (DPBS) without Ca^2+^ and Mg^2+^. Then, the cells were harvested with a) 0.25% trypsin EDTA (PAA Cell Culture Company^®^) for WEHI-164 cells and b) DPBS for IC-21 cells. Cells were counted using hemocytometer (Scepter^™^ 2.0 Cell Counter, Merck Millipore).

#### Evaluation of the impact of LLO on human leukemia cell lines

GFP-LLO was added to Jurkat cells culture in a 1:50 (v/v) ratio. The targeted cells were cultured (37°C, 5% CO_2_) in RPMI-1640|10%NCS medium for 24 hours. As a negative control (I), cells without addition of the LLO were cultured (37°C, 5%CO_2_) in RPMI-1640/10% NCS medium for 24 hours. The viability of cells was evaluated after 2 or 24 hours by flow cytometry using propidium iodide (Sigma, EU).

#### Evaluation of the impact of the designed system on human peripheral blood mononuclear cells

‘LLO nanocarriers’ (0.5×10^6^ ‘LLO nanocarriers’/ml) or ‘ligand modified LLO nanocarriers’ (0.5×10^6^ ‘ligand modified LLO nanocarriers’/ml) prepared according to the above procedures were cultured with human peripheral blood mononuclear cells (MNC). Designed platforms were added to the cells at concentration of 0.5×10^6^ platforms/ml in 1:50 (v/v) ratio (platform:cells). Targeted cells were maintained (37°C, 5% CO_2_) in culture medium RPMI-1640|10% NCS for 24 hours. The cells without the addition of the system were cultured as the standard negative control.

#### Evaluation of the impact of the designed system on WEHI-164 and IC-21 cells

‘LLO nanocarriers’ (0.5×10^6^ ‘LLO nanocarriers’/ml), ‘ligand modified LLO nanocarriers’ (0.5×10^6^ ‘ligand modified LLO nanocarriers’/ml) or the SNS (0.5×10^6^ SNS/ml) prepared according to the above procedures were cultured with WEHI-164 or IC-21 cells. Designed platforms were added to the cells at concentration of 0.5×10^6^ platforms/ml in 1:50 (v/v) ratio (platform:cells). Targeted cells were maintained (37°C, 5% CO_2_) in culture medium RPMI-1640|10% NCS for 24 hours.

The cells without the addition of the system were cultured as the standard negative control (control I). Simultaneously, additional controls were applied, i.e., cells cultured in the presence of platforms without incorporating LLO (bacteria|PEI|HA or bacteria|PEI|HA+TR) or the SNS. The presence of cells was assessed by flow cytometry using propidium iodide.

#### Flow cytometry

The presence of organisms was assessed using Canto II flow cytometer (Becton Dickinson Immunocytochemistry Systems, USA). The results were processed by the FACS Diva software system (Becton Dickinson, USA). Evaluated objects were separated from other events based on light scatter characteristics.

## Results and discussion

### Evaluation of the impact of the anti-tumor agent on eukaryotic cells

Active agents produced by microorganisms provided promising results in cancer therapy in recent years. Listeriolysin O (LLO) is a toxin from pathogenic bacterium *L*. *monocytogenes*. In its natural host, LLO is released into the cell endosome after phagocytosis as its major function is to facilitate *L*. *monocytogenes* intracellular survival. LLO exhibits the typical features of the cholesterol-dependent cytolysin with one major exception; it exhibits maximal activity in acidic pH, which is typically observed in the tumor environment [36]. Furthermore, the positive charge of the bacterial cytolysin can also facilitate its adhesion to the negatively charged cell membrane of the targeted cells. The anticancer properties of this factor have been previously studied in various cell lines, such as Jurkat cells or human peripheral blood mononuclear cells. Similar to other CDCs, LLO may display some specificity [34, 36]. However, it is necessary to further modify this toxin to ensure selectiveness towards cancer cells.

We have used and tested the LLO produced by genetically modified *E*. *coli* BL21 (DE3) cells. To facilitate the tracking process, we combined LLO with green fluorescence protein (GFP). The substantial modification of LLO with GFP (50% increase of molecular mass) did not lead to loss of its cytolytic activity when GFP was fused to the N-terminal part of LLO. GFP-LLO showed 97% activity of native LLO. In turn, LLO-GFP fusion protein displayed marginal activity (3%) therefore only active GFP-LLO variant was used in this work.

**Fig 1:**
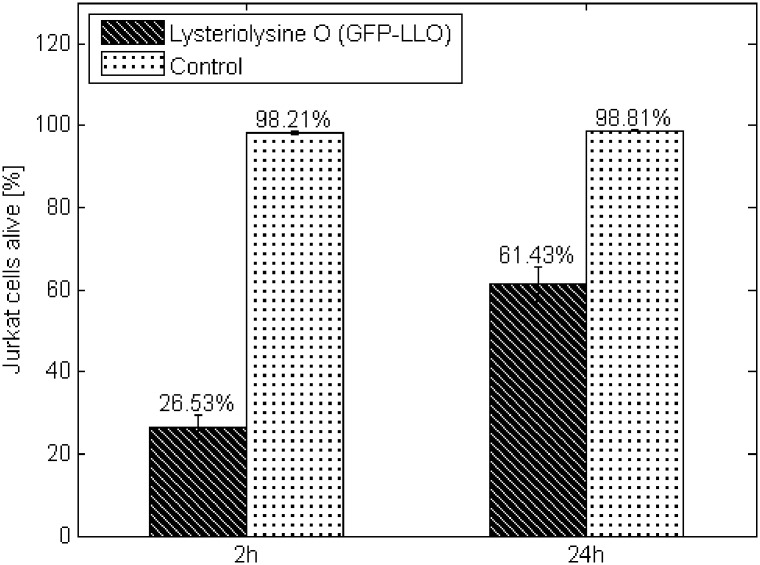
Jurkat cell viability during 24 hours of culture in the presence of listeriolysin O (GFP-LLO). Control—Jurkat cells cultured in the absence of listeriolysin O. The values are presented as the mean±SD.

We assessed the impact of GFP-LLO on eukaryotic cells during a 24-hour experiment. All experiments were performed in six repeatings. Fig 1 depicts overview of the experiment. Modified cytolysin was very effective during initial phase of experiment but cytolytic activity was significantly diminished during prolonged incubation. LLO is very sensitive to any physico-chemical changes in the environment [36] The loss of cytolytic activity is especially quick in physiological conditions for mammalian cells (pH 7.4 and 37°C) due to the irreversible protein unfolding [37].

**Fig 2:**
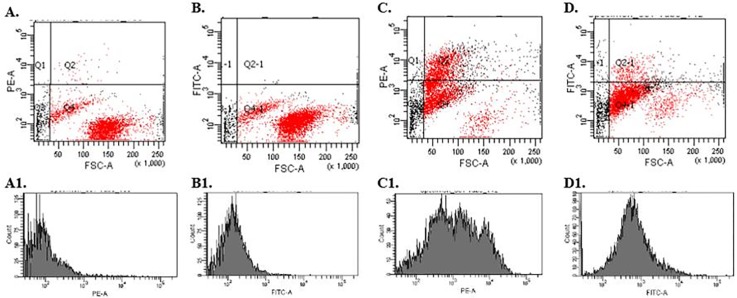
The example cytometric images obtained in flow cytometric analysis of Jurkat cells treated with GFP-LLO. The cells were analyzed after 2 hours of culture in the presence (C-D) or absence (A-B) of GFP-LLO. After incubation, cell viability was assessed using propidium iodide (PI) by flow cytometry. Dot plots represent cellular relative size (FSC). Histograms present the intensity of fluorescence in the PE-A and FITC-A channels, which correspond to signals from the respective dyes PI or GFP emission wavelength.

Fig 2 shows the representative plots obtained by flow cytometry analysis of human leukemia Jurkat cells cultured in the presence (Fig 2C and 2D) or absence (control group) (Fig 2A and 2B) of GFP-LLO for 2 hours. The dot plots contain the forward (FSC) parameter, which correlates with the relative size of the cells, whereas the histograms display the intensity of fluorescence in the PE-A and FITC-A channels, which correspond to signals from the respective dyes (propidium iodide—PI or green fluorescent protein—GFP) emission wavelength. Regarding the control group, the fluorescence signal of GFP was low (approximately 0.3%) and related to auto-fluorescence. On the contrary, high signals observed in the experimental group (approximately 9%) indicate that LLO (previously fused with GFP) was able to enter cells.

The impact of GFP-LLO on Jurkat cell viability was assessed using a membrane impermeable dye. PI binds to double-stranded DNA of dead cells that lack membrane integrity (observable as slight signals in the PI fluorescence histograms). The obtained results reveal a decline in the number of cells in the culture with GFP-LLO compared with the negative control (population cultured without the cytostatic agent). In the control, the percent of living cells was 99% (cytogram C, quadrant Q4), whereas it was approximately 63.9% in the experimental group (cytogram A, quadrant Q4.).

The results indicate that GFP-LLO exhibited lethal impacts on the studied cell line. The effect was visible after 2 hours of incubation and was sustained during the entire culture period. After 2 or 24 hours of incubation in the presence of GFP-LLO, the percent of living cells was reduced compared with negative control (population maintained without the cytostatic agent) (63% and 28%, respectively) (Fig 1).

### Analysis of HA|PEI nanothin membrane

The nanothin polyelectrolytes (PE) shell is the key element of the designed LLO system as it directly immobilizes the LLO. After preliminary studies with other membranes (e.g. modified alginate|polystyrene sulphonate, polylysine|polystyrene sulphonate [38]), we determined that the HA|PEI configuration is the most convenient for our applications due to its ability to sustain the bilayer structure. Both applied PEs, hyaluronic acid (HA) and poly(ethylenimine) (PEI), have been employed in biological and biotechnological applications. Being nontoxic and nonimmunogenic, HA has been used in nanoparticle preparations for the targeted delivery of anticancer factors to tumor cells through interaction with cell-surface HA receptors [39]. Consequently, HA application in our construct may be conductive to the system efficiency improvement, considering potential HA interaction with cell-surface HA receptors of targeted cells.

Moreover, due to its high biocompatibility, HA has recently gained popularity in the restrictive cosmetic industry. In contrast, PEI has been widely used as a material for carriers in cell transfection and as a coating shell for cell nanoencapsulation. Furthermore, HA and PEI exhibit good hydrophilicity.

**Fig 3:**
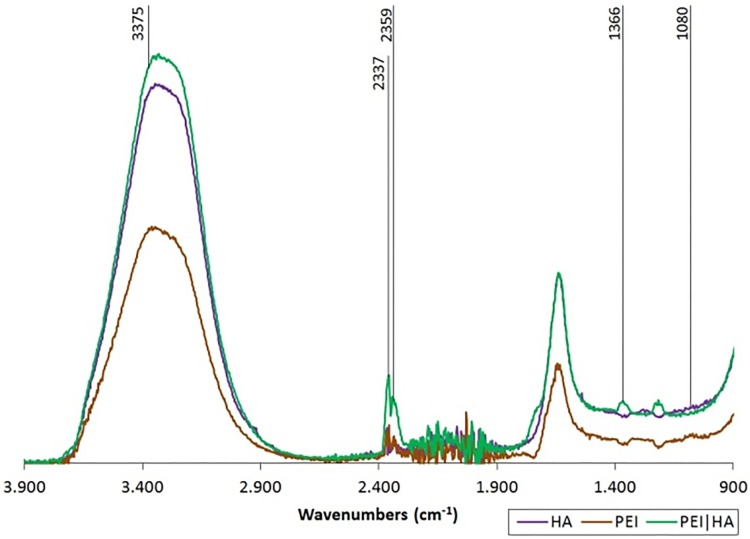
FTIR spectrum of HA, PEI and PEI|HA membrane.

To unambiguously validate the presence of the polyelectrolyte (PE) shells on the substrate, we used atomic force microscopy (AFM) and Fourier transform infrared spectroscopy (FTIR). FTIR signals were evaluated for all of the following studied layers: hyaluronic acid (HA), poly(ethylenimine) PEI and the combination (HA|PEI). The presence of membranes was indirectly demonstrated by the detection of characteristic picks. Signals were noted at the following frequencies [cm-1]: 3375 exhibiting N-H stretching vibrations in PEI and HA; 2359, 2337 exhibiting N-H vibrations of hydrogen interactions in PEI and HA; 1366 exhibiting C-CH_3_ presence and 1080 attributed to C-N stretching vibrations in PEI and HA (Fig 3).

**Fig 4:**
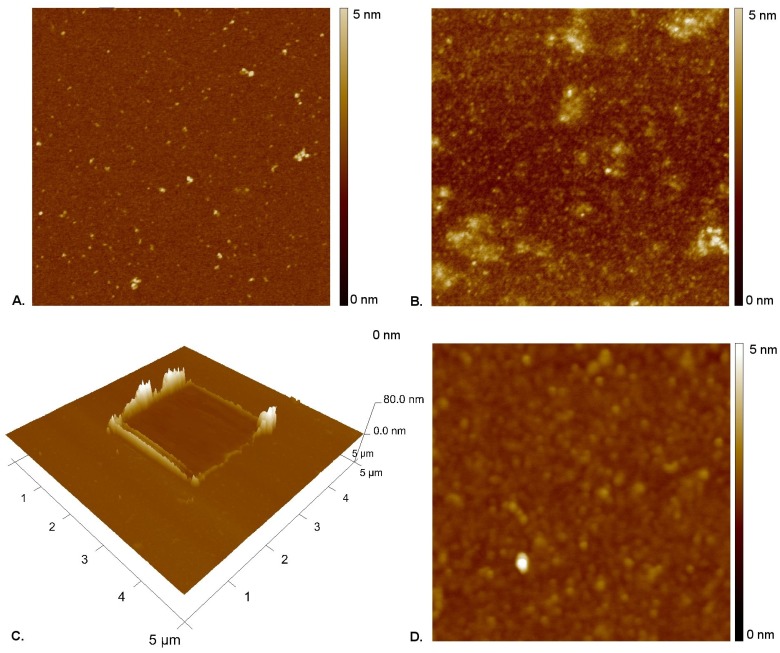
AFM visualization of HA and PEI layers deposited on the gold mica substrate cover. (A) HA layer on the gold mica substrate cover, (B) PEI layer on the gold mica substrate cover, (C) Example of AFM scratching experiment on PEI/HA film, (D) morphology of PEI/HA film.

To visualize the morphological structure of the polyelectrolyte shells, we applied the AFM technique. Both HA and PEI layers were deposited on mica substrate. The obtained data are presented in Fig 4(A)–4(D). In branch structured PEI, several PE centers scattered over the surface are visible (Fig 4B). On the contrary, in the substrate covered with HA of smaller molecular weight, uniform morphology of the surface is observed (Fig 4A).

The AFM scratching method let us determine the thickness of membrane formed by PEI/HA system equal to 3 nm by analyzing the difference of height between the film and revealed surface of a solid substrate. The PEI/HA film show good homogeneity which is visible in large (Fig 4C) and small (Fig 4D) scale pictures.

The measure of interaction between the polyelectrolyte layers is the work of adhesion calculated by an integration of force—distance dependencies according to the following formula:

$$W_{ad}=\int F_{ad}dz$$ (1)

where *F*_*ad*_ is adhesion force and *z* is the distance of sphere from a surface. The calculated average work of adhesion occurring between layers PEI and HA value was equal to 4.32±1.34×10^−15^ J (n = 4).

The HA|PEI configuration is the most convenient for our platform due to its ability to sustain the bilayer structure because of relatively high work of adhesion between the layers as compared with interaction between PEI and weak polyelectrolytes [40].

The cell cores (IC-21 cells) coated with PE membrane were used to estimate the applied membrane molecular weight cut-off value. Diffusive permeability was evaluated using a thermodynamic description of diffusive mass transport across a homogenous membrane (Fick’s law) and a two-compartment model [41]. The dextrans were used as the model particles. The PEI|HA membrane cut-off value was assessed at the 150 kDa level, which is sufficient for transport of listeriolysin through the membrane wall. The diffusion coefficient of the shell at a mean 3 nm thickness built of PEI|HA membrane for Dextran 150 was 95.167×10^−12^ [cm^2^×s^-1^].

To evaluate the wettability of PEI|HA membrane on an alginic support, we used the surface energy analyzer. The value of the contact angle measured for the dedicated layer was 34.68±2.45 [°], indicating the relatively high hydrophilicity of the designed membrane system.

### Design of the systems for listeriolysin O targeted delivery

To identify the most effective system for anti-tumor agent delivery, we designed and examined three different platforms. We studied the following systems: ‘LLO nanocarrier’, the basic platform consisting of the bacterial core nanocoated with a polyelectrolyte bilayer with active agent immobilized within; ‘ligand modified LLO nanocarrier’, ‘LLO nanocarrier’ modified by biotin-avidin-biotinylated transferrin complexes and ‘stabilized nanosystem’ (SNS) build with the stabilizing element bearing transferrin (‘cell core’) and ‘LLO nanocarriers’ coupled to the cell core via a biotin-avidin-biotin bridge.

**Fig 5:**
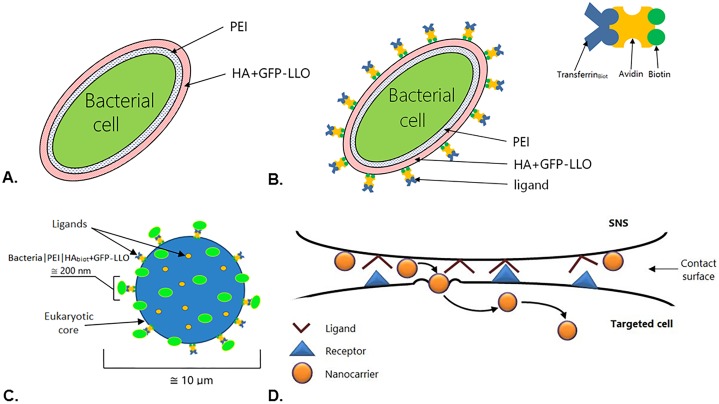
The construction of (A) ‘LLO nanocarrier’, (B) ‘ligand modified LLO nanocarrier’ and (C) SNS. (D)The model of the contact surface between the SNS and the targeted cell. Bacterial cells nanocoated with a polyelectrolyte bilayer (poly(ethylenimine) and hyaluronic acid with GFP-LLO) unmodified (A) or modified (B) by biotin-avidin-biotinylated transferrin complex; ligand modified cell cores (stabilizing element) with biotynylated LLO nanocarriers (C).

To prepare the ‘LLO nanocarrier’ (Fig 5A), we coated bacterial cells with the nanothin PEI|HA bilayer. LLO was immobilized within the external (hyaluronic acid) layer of the system. Additionally, we improved the ‘LLO nanocarrier’ by transferrin incorporation. The glycoprotein was immobilized within the extraneous shell of the system, forming the ‘ligand modified LLO nanocarrier’ (Fig 5B).

The construction of the third platform—the ‘stabilized nanosystem’ (SNS)–consisted of two main steps. First, transferrin ligands were anchored to the surface of the ‘cell core’. Second, the prepared ‘cell core’ was bound with the biotinylated ‘LLO nanocarriers’. The SNS were obtained as a result (Fig 5C).

Cells can absorb small elements by engulfing them in the process called phagocytosis. Accordingly, in the designed systems, we applied bacteria, the size of which allows adsorption by cells. Moreover, to prevent the destruction of the active agent during the experiment, the agent was immobilized within the PEI|HA bilayer covering the bacterial core.

To boost the stability of the prepared LLO platform and intensify the effect towards targeted cells, we used a stabilizer, the eukaryotic cell core, to extend the system. Accordingly, previously studied ‘LLO nanocarriers’ were coupled to the ‘cell core’ via a biotin-avidin-biotin bridge. In addition, to facilitate cytostatic agent delivery to tumor cells, transferrin ligands were anchored to the surface of the cell core. The application of the stabilizer increases the final dose of delivered active substance and simultaneously reduces its unnecessary dissipation throughout body fluids. We selected a core of biological origin to minimize the extent of the platform and maintain non-immunogenicity of the system.

Numerous tumor-associated receptors, including transferrin receptor, asialoglycoprotein receptor, and estrogen receptor, have been investigated using receptor-targeting approaches [42–44]. We concentrated on transferrin receptors that exhibit increased expression on the surface of proliferating tumor cells. Consequently, platforms containing transferrin molecules increase the affinity for cancer cells. Therefore, to enhance the system selectivity and facilitate its direct delivery to the target, we modified the surface of ‘LLO nanocarriers’ using transferrin. A model of the contact surface between the designed the SNS and targeted cells is presented in Fig 5D.

**Fig 6:**
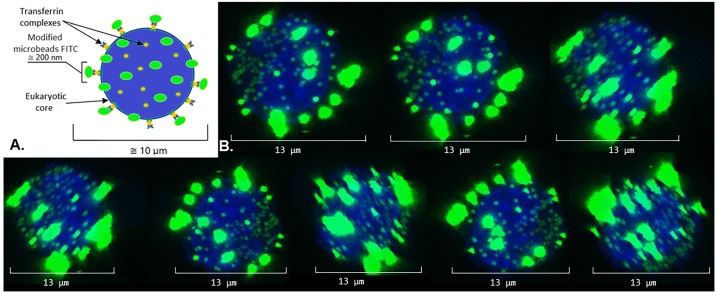
(A) The model of the SNS applied for visualization. (B) Visualization of the SNS. (A) Blue circle—stabilizer (‘cell core’), green ellipses—modified microbeads; yellow circles—transferrin complexes anchored to the eukaryotic cell membrane. (B) Series of images taken during system rotation around its axis. Green fluorescence—FITC stained elements (microbeads FITC, anti-transferrin FITC antibody); blue fluorescence—stabilizer—eukaryotic nuclei stained with Hoechst 33342.

After confirming nanocarriers coupling with the ‘cell core’, we applied confocal microscopy. To facilitate the analysis, we used FITC-labeled microbeads coated with a PEI|HA bilayer instead of non-fluorescent bacterial carriers. To detect transferrin, an anti-transferrin FITC antibody (Sigma, EU) was used. Microscopic observations are presented in Fig 6. We observe the blue fluorescence of nuclei of the cell core and intensive homogeneous green fluorescence of the encapsulated by PE microbeads attached to the cell core surface. Furthermore, smaller green dots represented transferrin complexes with the anti-transferrin FITC antibody are visible on the cell core surface. These results demonstrate the binding between the ‘cell core’ and model nanocarriers. Moreover, the data indicate the coupling of transferrin complexes with eukaryotic cells.

### Confirmation of the platform elements

The presence of the basic platform—LLO nanocarriers was confirmed using flow cytometry. Coating of the non-fluorescent bacterial core with polyelectrolyte layer with immobilized GFP-LLO resulted in obtaining meanly 86.9±5.3 FITC positive events.

**Fig 7:**
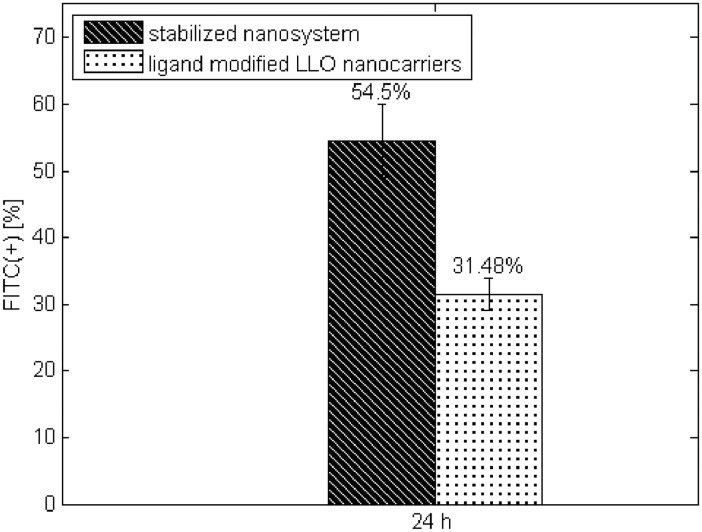
Evaluation of FITC expression of modified nanocarriers LLO or the SNS. The values are presented as the mean±SD.

The presence of the individual elements of the designed systems, especially the polyelectrolyte layer, was confirmed indirectly by immunocytochemical reaction with an anti-transferrin antibody. Accordingly, we incorporated transferrin complexes within the HA layer of ‘ligand modified LLO nanocarrier’ or the ‘cell core’ of the SNS. After 24 hours of incubation in a culture medium, we assessed the formation of complexes using an anti-transferrin antibody (FITC-positive events) via flow cytometry. All experiments were performed in six repeatings. As shown in Fig 7, the percent of FITC-positive events in the examined SNS was increased (54%) compared with ‘ligand modified LLO nanocarrier’ (31%). The obtained data suggest that the stabilized nanosystem provides a potentially increased extent of ligands compared with non-stabilized platforms.

**Fig 8:**
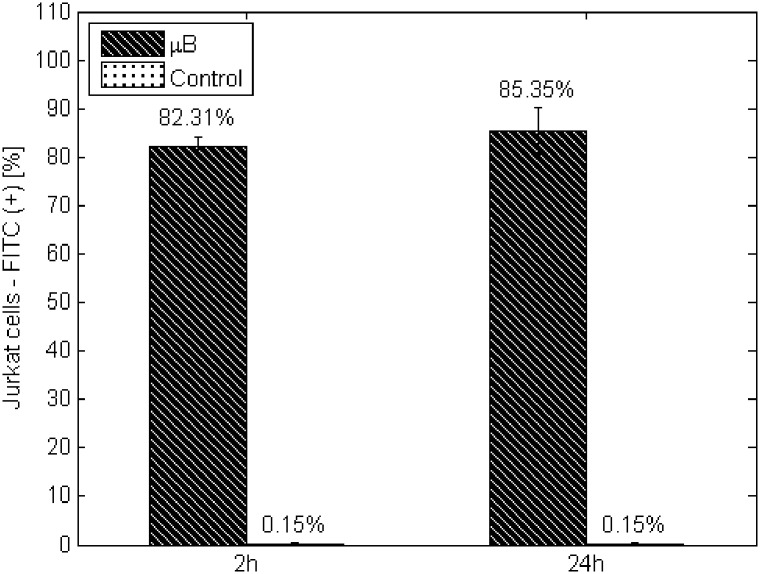
Evaluation of FITC-positive events for Jurkat cells during 24 hours of culture in the presence of microbeads coated by PEI|HA layer. Control—Jurkat cells culture without additives. The values are presented as the mean±SD.

To examine the trace of nanocarrier interaction with the target cells, we also applied flow cytometry. To facilitate analysis, we substituted the bacterial cores of LLO nanocarriers with FITC-labeled microbeads (~200 nm diameter). Then, Jurkat cells were cultured in the presence of those ‘LLO nanocarrier’ platforms (experimental group) for 24 hours for flow cytometry evaluation. The intensity of fluorescence in the FITC-A channel corresponding to the signals from the FITC emission wavelength was evaluated. In the experimental group, the fluorescence signal of FITC was relatively high (approximately 84%) compared with the control group (cells cultured without the “LLO nanocarrier”) at approximately 0.2% (Fig 8). The obtained results demonstrate uptake of 200-nm particles into the cell. We assume that the interaction between the targeted cell and the SNS proceeded via a similar mechanism. First, facilitated by avidity to the targeted cells, the SNS adhered to the cell surface. Then, the ‘LLO nanocarriers’ initially bioconjugated to the stabilizer (‘cell core’) undergo cellular uptake.

**Fig 9:**
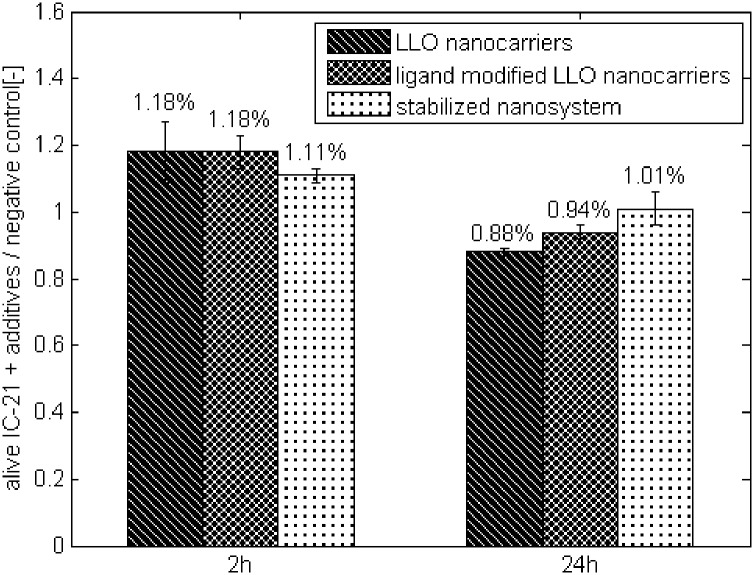
IC-21 cell viability during 24 hours of culture in the presence of the SNS, ‘ligand modified LLO nanocarriers’ and ‘LLO nanocarriers’ compared with the negative control. Control—culture of IC-21 cells. The values are presented as the mean±SD.

To further assess whether the LLO molecules are adsorbed by cells via phagocytosis, we examined the impact of all constructed systems on cells exhibiting phagocytic functionality, namely the macrophage cell line IC-21 (mouse macrophage). No significant difference in cells viability was noted between the macrophage cells incubated in the presence of designed systems and negative control (population cultured without the system) during 24 hours of culture (Fig 9). These data confirm that the platforms do not exert a lethal impact on evaluated cells.

### Comparison of the effectiveness of ‘LLO nanocarriers’, ‘ligand modified LLO nanocarriers’ and ‘stabilized nanosystem’

**Fig 10:**
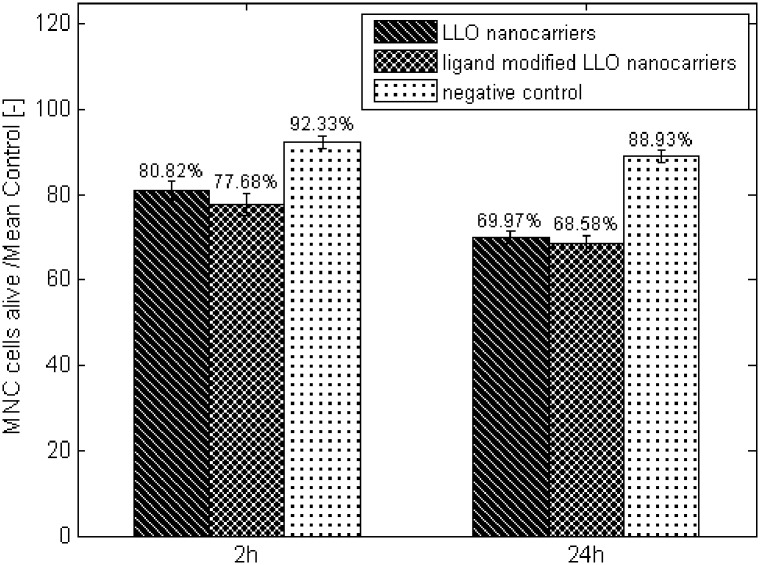
MNC cell viability during 24 hours of culture in the presence of ‘ligand modified LLO nanocarriers’ or ‘LLO nanocarriers’. Control—culture of MNC cells. The values are presented as the mean±SD.

Assessing the influence of the basic ‘LLO nanocarriers’ system or ‘modified LLO nanocarriers’ on human peripheral blood mononuclear cells there was no significant difference in the percentage of viable cells cultured in presence of ‘modified LLO nanocarriers’ compared with culture in presence of ‘LLO nanocarriers’ after two hours (p = 0.0516>0.05) or after 24 hours (p = 0.1637>0.05). However a significant difference in the percentage of viable cells in the group cultured with ‘LLO nanocarriers’ or ‘modified LLO nanocarriers’ compared with control (the cells without the addition of the systems) was observed after two hours (p = 0.0001<0.05) or after 24 hours (p = 0.0001<0.05) or after 24 hours (p = 0.0000<0.05) or after 24 hours (p = 0.0000<0.05) (Fig 10).

It can be caused by lower transferrin receptors expression compared with actively growing cells of cell lines [45].

Consequently, for further examinations the WEHI 164 cells were applied. All experiments were performed in six repeatings.

**Fig 11:**
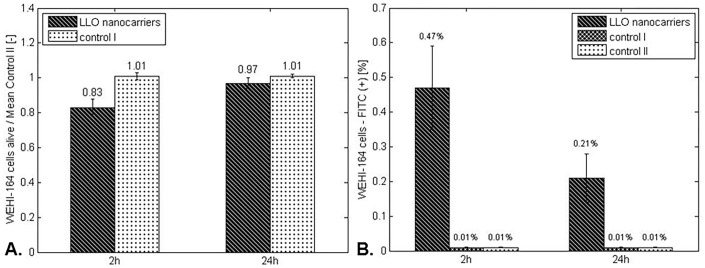
A. WEHI-164 cell viability during 24 hours of culture in the presence of ‘LLO nanocarriers’. B. The percentage of FITC-positive events for WEHI-164 cells during 24 hours of culture in the presence of ‘LLO nanocarriers’. Control I—WEHI-164 cells cultured in the presence of ‘LLO nanocarriers’ without incorporated GFP-LLO. Control II—culture of WEHI-164 cells. The values are presented as the mean±SD.

Assessing the influence of the basic system (‘LLO nanocarriers’) on WEHI-164 cells, a significant difference in the percentage of viable cells in the experimental group compared with control I (population cultured in the presence of ‘LLO nanocarriers’ without incorporated GFP-LLO) was observed. Moreover, for ‘LLO nanocarriers’, the percentage of FITC-positive events in the experimental group after 24 hours of culture were reduced 3-fold compared with the culture examined after 2 hours of incubation, revealing GFP-LLO loss related to cell necrosis (Fig 11).

**Fig 12:**
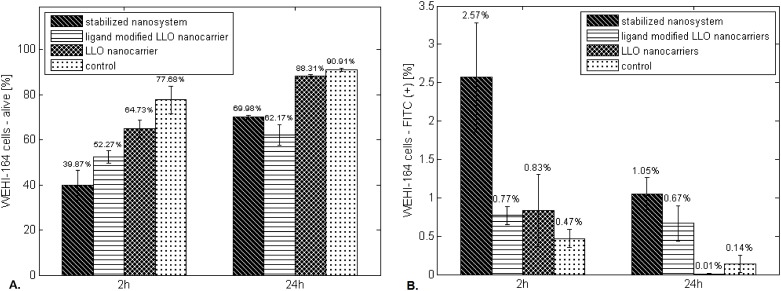
A. WEHI-164 cell viability during 24 hours of culture in the presence of the SNS, ‘ligand-modified LLO nanocarriers’ and ‘LLO nanocarrier’. (B) The percent of FITC-positive events for WEHI-164 cells during 24 hours of culture in the presence of the SNS, ‘ligand-modified LLO nanocarriers’ and ‘LLO nanocarrier’. (A) Significant differences in cell viability compared with negative control between the culture with ‘LLO nanocarriers’ (p = 0.0010<0.05), ‘ligand-modified LLO nanocarriers’ (p = 0.0000<0.05) or SNS (p = 0.0002<0.05) after 2-hour of culture. (B) Control—culture of WEHI-164 cells. The values are presented as the mean±SD.

To evaluate the influence of the ‘LLO nanocarriers’, ‘ligand modified LLO nanocarriers’ and SNS platforms on eukaryotic cells, we cultured WEHI-164 cell line in their presence. We observed a mean 35% decline in viable cells after 2 hours of incubation in the presence of ‘LLO nanocarriers’, a 48% decline in the presence of ‘ligand modified LLO nanocarriers’ and a 60% decline for the SNS. All examined systems exerted lethal effects on the evaluated cells. A significant difference in cell viability was noted between the negative control (WEHI-164 cultured alone) and each one of the experimental groups after 2 hours of culture (Fig 12). Nevertheless, the SNS demonstrated the highest impact on WEHI-164 cells during 2-h incubation. We observed a significant difference in the percentage of viable cells cultured with the SNS compared with culture in the presence of the ‘LLO nanocarriers’ (p = 0.0000<0.05), the ‘ligand modified LLO nanocarriers’ (p = 0.0041<0.05), or the negative control (p = 0.0002<0.05).

Additionally, the lethal impact of the ‘ligand modified LLO nanocarriers’ and the SNS was maintained up to 24 hours. Statistical differences in cell viability was noted between the cells cultured with the SNS compared with the ‘LLO nanocarriers’ (p = 0.0002<0.05) or the negative control (p = 0.0010<0.05) and between the cells cultured with the ‘ligand modified LLO nanocarriers’ compared with the ‘LLO nanocarriers’ (p = 0.0000<0.05) or the negative control (p = 0.0000<0.05). Importantly, there were the following two negative controls for each experiment: WEHI-164 cultured alone and the population cultured with the respective system without LLO incorporation.

To estimate whether GFP-LLO delivered by designed platforms actually interacted with the cells, we evaluated the percent of FITC-positive events corresponding to signals from the GFP emission wavelength.

Fig 12 presents results obtained for the control and experimental groups during 24 hours of culture. The fluorescent signals are not visible for the control group. Moreover, we observed a significant difference between the signal obtained from the SNS and other systems (p<0.05) after 2 hours of culture. The number of events with fluorescent signals declined after 24 hours compared with 2 hours in all applied platforms, which is caused by cell necrosis.

The systems bearing transferrin (‘ligand modified LLO nanocarriers’ and SNS) demonstrated increased lethal effects on targeted cells after 2 hours of culture compared with the non-modified nanocarrier (‘LLO nanocarrier’). These results indicate that the presence of this protein supports the system’s effectiveness. As previously mentioned, proliferating tumor cells exhibit increased expression of transferrin receptors on their surface; thus, the platform with incorporated transferrin exhibits increased affinity towards tumor cells. Accordingly, a higher number of LLO nanocarriers reach the target, which increases the platform efficiency.

## Conclusions

After examining three different platforms, we conclude that the SNS is the most effective platform. Based on biologically derived elements, the unique design of the developed system supplies LLO to the targeted cells. The constructed system provides immediate cytotoxic effects on tumor cells while simultaneously ensuring the protection of the active agent from potential destruction during the experiment. Comparing the performance of constructed platforms in targeted cells, the increased lethal effects of systems bearing transferrin is observed, indicating that the ligand increases the platform affinity towards the tumor cells. Finally, the ‘cell core’ with transferrin ligands ensures system stability and enhances the titer of the LLO nanocarriers that are delivered to the targeted cells. Of note, the macromolecular substances involved because of phagocytosis induced visible intracellular effects after a few dozen minutes. This process cannot be observed in cases of macrophages because of their phagocytic function. The unique advantage of the system is its possible application in local listeriolysin O anti-tumor therapies. These experiments provided the basis for the further development of LLO delivery systems as an alternative to targeting chemotherapeutic drugs in local anti-tumor therapy.
